# Incidence and Outcomes of Hemorrhagic Stroke among Adults in Spain (2016–2018) According to Sex: A Retrospective, Cohort, Observational, Propensity Score Matched Study

**DOI:** 10.3390/jcm10163753

**Published:** 2021-08-23

**Authors:** Jose M. de Miguel-Yanes, Ana Lopez-de-Andres, Rodrigo Jimenez-Garcia, Valentin Hernandez-Barrera, Javier de Miguel-Diez, Manuel Méndez-Bailón, Napoleón Pérez-Farinós, Nuria Muñoz-Rivas, David Carabantes-Alarcon, Marta López-Herranz

**Affiliations:** 1 Internal Medicine Department, Hospital General Universitario Gregorio Marañón, Instituto de Investigación Sanitaria Gregorio Marañón (IiSGM), Universidad Complutense de Madrid, 28040 Madrid, Spain; josemaria.demiguel@salud.madrid.org; 2 Department of Public Health & Maternal and Child Health, Faculty of Medicine, Universidad Complutense de Madrid, 28040 Madrid, Spain; rodrijim@ucm.es (R.J.-G.); dcaraban@ucm.es (D.C.-A.); 3 Preventive Medicine and Public Health Teaching and Research Unit, Health Sciences Faculty, Universidad Rey Juan Carlos, Alcorcón, 28922 Madrid, Spain; valentin.hernandez@urjc.es; 4 Respiratory Department, Hospital General Universitario Gregorio Marañón, Instituto de Investigación Sanitaria Gregorio Marañón (IiSGM), Universidad Complutense de Madrid, 28040 Madrid, Spain; javier.miguel@salud.madrid.org; 5 Internal Medicine Department, Hospital Universitario Clínico San Carlos, Universidad Complutense de Madrid, 28040 Madrid, Spain; manuel.mendez@salud.madrid.org; 6 Public Health and Psychiatry Department, Faculty of Medicine, Universidad de Málaga, 29010 Málaga, Spain; napoleon.perez@uma.es; 7 Internal Medicine Department, Hospital Universitario Infanta Leonor, 28031 Madrid, Spain; nmunozr@salud.madrid.org; 8 Nursing Department, Faculty of Nursing, Physiotherapy and Podology, Universidad Complutense de Madrid, 28040 Madrid, Spain; martal11.ucm@gmail.com

**Keywords:** hemorrhagic stroke, sex differences, decompressive craniectomy, in-hospital mortality

## Abstract

(1) Background: We aim to analyze sex differences in the incidence, clinical characteristics and in-hospital outcomes of hemorrhagic stroke (HS) in Spain (2016–2018) using the National Hospital Discharge Database. (2) Methods: Retrospective, cohort, observational study. We estimated the incidence of HS in men and women. We analyzed comorbidity, treatments, procedures, and hospital outcomes. We matched each woman with a man by age, type of HS and medical conditions using propensity score matching. (3) Results: HS was coded in 57,227 patients aged ≥18 years (44.3% women). Overall, men showed higher incidence rates (57.3/10^5^ vs. 43.0/10^5^; *p* < 0.001; IRR = 1.60; 95% CI: 1.38–1.83). Women suffered more subarachnoid hemorrhages (25.2% vs. 14.6%), whereas men more often had intracerebral hemorrhages (55.7% vs. 54.1%). In-hospital mortality (IHM) was higher for intracerebral hemorrhage in both men and women. Women underwent decompressive craniectomy less often than men (5.0% vs. 6.2%; *p* < 0.001). After matching, IHM among women was higher (29.0% vs. 23.7%; *p* < 0.001). Increments in age, comorbidity and use of anticoagulants and antiplatelet agents prior to hospitalization were associated were higher IHM, and decompressive craniectomy was associated with lower IHM in both sexes. After multivariable adjustment, women had higher IHM (OR = 1.23; 95% CI: 1.18–1.28). (4) Conclusion: Men had higher incidence rates of HS than women. Women less often underwent decompressive craniectomy. IHM was higher among women admitted for HS than among men.

## 1. Introduction

Hemorrhagic stroke (HS) is a devastating medical condition with a high short-term mortality. In a review published in 2010, which included 36 population-based studies, the authors reported an incidence of 25 cases of HS per 100,000 inhabitants, with non-significant differences in the incidence between men and women [1]. However, other studies have generally found a higher overall incidence in men [2]. Most authors agree that incidence rates have remained relatively constant over time in developed countries [3], but they seem to have been growing in developing countries [4].

Very often, a distinction is not made between the subtypes of hemorrhagic stroke (e.g., intracerebral hemorrhage or subarachnoid hemorrhage), and some research exclusively evaluated cases of parenchymal bleeding. Subarachnoid hemorrhage is believed to be more common among women [5]. Therefore, lack of inclusion of all the subtypes of HS may yield apparently disparate numbers as far as incidence is concerned. Apart from the location of the bleeding, sex may influence the development of perihematomal edema, and treatment of the hemorrhagic stroke or its complications may differ according to sex [6,7].

In the review by van Asch et al., mortality during the first month after the event ranged from 16% to more than 50%, with a median value around 40% [1]. Gokhale et al., underscored additional limitations of previous research work: many reports were from before 2000, some of the studies treated intracerebral hemorrhage as a stroke subtype in a total stroke population instead of considering it as a distinct entity, and relatively few studies included significant numbers of cases [8], albeit it is common to adjust for age and functional status [9]. Propensity score matching (PSM) is a method that makes populations with a different distribution of baseline variables associated with hospital outcomes more comparable and, therefore, reduces residual confounding [10].

Here we used PSM to assess the sex-related differences in the incidence, clinical characteristics, use of decompressive craniectomy and in-hospital mortality for patients admitted with a primary diagnosis of HS to Spanish hospitals from 2016 to 2018. We also used multivariable regression to identify which of the study variables were independently associated with in-hospital mortality (IHM) for women and men hospitalized with HS.

## 2. Materials and Methods

### 2.1. Study Design and Data Source

We conducted a retrospective observational cohort study based on the data collected by the Spanish National Hospital Discharge Database (SNHDD). This registry provides basic demographic data, up to twenty diagnoses and procedures, and the outcome for each hospitalization in any Spanish hospital. Since the year 2016, the International Classification of Disease version 10 (ICD-10) is being used for coding by the SNHDD. Detailed information on the SNHDD is available online [11].

### 2.2. Study Population

We analyzed data from adults (aged 18 years or over) registered by the SNHDD from 1 January 2016 to 31 December 2018. Only subjects discharged with a primary diagnosis of HS were included in the study population. The ICD-10 codes for HS are shown in Table S1. For study purposes, we grouped HS in three subtypes: “Nontraumatic subarachnoid hemorrhage”, “Nontraumatic intracerebral hemorrhage” and “Other and unspecified nontraumatic intracranial hemorrhage”.

As we do not have information on the characteristics of any previous stroke, all those patients with a code for history of previous stroke (ICD-10 code Z86.73) were excluded from the study population.

### 2.3. Study Variables

Our main study variables were the incidence of HS, the use of decompressive craniectomy and the IHM. Incidence rates were calculated using the Spanish populations by year, sex and age groups, provided by the Spanish National Statistics Institute as described before [12,13]. Patients were classified as receivers of a decompressive craniectomy if any of the codes for this procedure (Table S1) appeared in any of the 20 procedure fields. Secondary study variables included age, cardiovascular risk factors, alcohol abuse, comorbid conditions, the use prior to hospital admission of oral anticoagulants (OACs) and antiplatelet agents, receipt of mechanical ventilation (invasive and non-invasive) and length of hospital stay (LOHS). The codes for these conditions, treatments and procedures are shown in Table S1.

Comorbidity was assessed using the Charlson’s comorbidity index (CCI) as categorized in three groups “CCI = 0”, “CCI = 1” and “CCI > 1”. The ICD-10 codes to identify the CCI conditions were those described by Sundararajan et al. [14]. Additional variables accounted for were atrial fibrillation, anemia, depression, sepsis and nosocomial pneumonia (ICD-10 codes shown in Table S1).

### 2.4. Matching Method

PSM analysis was applied to control for the confounding effect of sex differences in the baseline characteristics that can be associated with our main study variables. The PSM model was constructed including type of HS (up to the last two decimals), age, cardiovascular risk factors and other conditions present on admission. Details regarding the PSM approach with the SNHDD have been described elsewhere [15].

### 2.5. Statistical Analysis

All results are presented separately for women and men. To estimate differences in the incidences according to sex and age groups, we constructed age-adjusted Poisson regression models.

We report means with standard deviation (SD), or medians with interquartile range (IQR) for age/CCI and LOHS, respectively. For all the remaining variables, absolute frequencies and percentages are shown.

Mean values between men and women were compared using the t-test, or the Mann–Whitney test, depending on the homogeneity of the variance (Levene’s test). Differences in percentages were assessed using the chi-square test. We constructed multivariable logistic regression models to identify the variables associated with IHM among men and women. Details on model construction have been provided elsewhere [15].

### 2.6. Sensitivity Analysis

To confirm the results of the PSM to assess the sex-related differences in the IHM, we complemented the statistical analysis creating multivariable logistic regression models including the entire study population and considering sex as the main outcome variable.

The PSM process and all bivariate and multivariable analyses were conducted using Stata version 14 (Stata, College Station, TX, USA). For all comparisons, statistical significance was set at a *p* value < 0.05 (two-sided).

### 2.7. Ethical Aspects

This study was conducted using secondary administrative data that can be obtained on request to the Spanish health authorities by any investigator [16]. All personal identifiers were deleted before the databases were provided to us, so confidentiality was fully ensured. Therefore, and according to the Spanish laws, it is unnecessary to present the study protocol for evaluation to an ethics committee.

## 3. Results

From 2016 to 2018, a total of 57,227 patients aged ≥18 years were discharged from Spanish hospitals with a primary diagnosis of HS. Men accounted for 55.7% (*n* = 31,868) and women for 44.3% (*n* = 25,359).

### 3.1. Incidence of Hemorrhagic Stroke According to Sex

The global incidence of HS per 100,000 inhabitants was higher among men (57.3/10^5^) than among women (43.0/10^5^; *p* < 0.001) (Table 1), and differences in the same direction were seen in all age strata. For both sexes, incidences increased with age, reaching the highest values in people 85 years old or over (346.3/10^5^ and 213.0/10^5^ in men and women, respectively). Using age-adjusted Poisson regression analyses, we obtained an incidence rate ratio (IRR) of 1.60 (95% confidence interval (CI): 1.38–1.83). This means that in the period 2016/2018 men had a 60% greater incidence of hospitalizations for HS than women did.

### 3.2. Clinical Characteristics and Hospital Outcomes According to Sex

The distribution according to type of HS, age, CCI, cardiovascular risk factors, lifestyle variables and use of OACs and antiplatelet agents before and after PSM for men and women are shown in Table 2. Mean ages were 72.7 years old for women and 70.2 years for men (*p* < 0.001). Men were overrepresented in the younger age groups and women in the older ones.

We found significant sex differences for the type of HS. Women suffered more subarachnoid hemorrhages (25.2% vs. 14.6%), whereas men were more often admitted for intracerebral hemorrhages (55.7% vs. 54.1%) and unspecified intracranial hemorrhages (29.7% vs. 20.7%) (all *p*-values < 0.001). The mean number of comorbidities, according to the CCI, was higher among men than among women (0.85 vs. 0.67; *p* < 0.001). Men had a code for hypertension and for alcohol abuse more frequently than women did.

The use of OACs (13.71% vs. 13.82%; *p* = 0.718) was similar in both sexes whereas use of antiplatelet agents was significantly more prevalent among men (10.08% vs. 7.82%; *p* < 0.001).

Men suffered most comorbid conditions analyzed with a significantly higher prevalence than women, exceptions made for dementia, rheumatoid disease, anemia and depression, which were more prevalent among women (Table 3). Women underwent decompressive craniectomy less often than men did: 5.0% vs. 6.4% before PSM (*p* < 0.001), and 5.0% vs. 6.2% after PSM (*p* < 0.001). LOHS and IHM showed significantly worse figures for women than for men. After PSM, IHM among women admitted for a HS was over 5 points higher than among men (29.0% vs. 23.7%; *p* < 0.001).

### 3.3. Factors Associated with IHM during Admission for Hemorrhagic Stroke

According to the type of HS, IHM was highest for nontraumatic intracerebral hemorrhage in both men and women (Table 4). For all types of HS, IHM was higher among women than among men, both before and after PSM. Increments in age and CCI were associated were increased IHM in both sexes, with women showing higher IHM for most categories of these variables. Use of OACs and antiplatelet agents were associated with higher IHM among women than among men.

For most chronic conditions, we detected no significant differences in IHM between men and women, either before or after PSM (Table 5). However, women with diabetes, hemiplegia/paraplegia, chronic renal disease and atrial fibrillation did not survive to hospital discharge in a higher proportion than that for men. Decompressive craniectomy was associated with a higher IHM among women than among men both before and after PSM (20.76% vs. 15.64% and 20.76% vs. 14.26%, respectively; both *p*-values < 0.001).

The results of the multivariable logistic regression analyses are shown in Table 6. For men and women, IHM increased with age, congestive heart failure, dementia, diabetes, sepsis, use of OACs or antiplatelet agents and the need for mechanical ventilation during hospitalization. COPD, kidney disease and peripheral vascular disease predicted IHM only among men.

Obesity was associated with lower IHM both among women and men (OR = 0.87, 95% CI 0.82–0.93 and OR = 0.88, 95% CI 0.83–0.93, respectively). Undergoing decompressive craniectomy was significantly associated with survival at hospital discharge in both sexes (OR = 0.49, 95% CI 0.42–0.58 in women and OR = 0.38, 95% CI 0.33–0.43 in men).

### 3.4. Sensitivity Analysis

When we joined the databases of women and men for a sensitivity analysis, we confirmed the results of the PSM, finding that women were 23% significantly more likely to die in the hospital when admitted for HS than men were (OR = 1.23, 95% CI 1.18–1.28) (Table 6).

## 4. Discussion

Here we found that the incidence of HS per 100,000 inhabitants was higher among men than among women. Women more often suffered subarachnoid hemorrhages, whereas men were more often admitted for intracerebral and other unspecified intracranial hemorrhages. Women underwent decompressive craniectomy less often than men even though decompressive craniectomy was associated with increased survival at hospital discharge in both sexes. Obesity was associated with lower IHM both among women and men, and the use of OACs or antiplatelet agents was associated with higher IHM. In the multivariable regression analysis, women were 23% more likely than men to die in the hospital when admitted for HS.

In our study, the incidence of HS was higher among men than among women. Similar differences have been reported in previous research [2,17,18]. Studies including different subtypes of HS generally include more cases of cerebral parenchymal bleeding than those with subarachnoid hemorrhage. The latter is more commonly coded in women, but its impact on the overall number of cases of HS is lesser. We detected a higher number of HS in women ≥85 years old than in men of the same age group, but the incidence per 100,000 people was lower. Previous publications have reported higher numbers of HS in very old women; they did not account for the total population in the denominator [19]. Thus, an age interaction effect is unlikely to be proven.

Several factors may help explain the higher incidence of HS in men. At a given age, men have higher blood pressure than women [20], which is a paramount factor for the onset of brain bleeding [21]. It is not known whether gonadal hormones may be playing a role, but different patterns of HS have been described for premenopausal as compared with postmenopausal women [22]. Estrogen treatment results in decreased cell death in neuronal cultures after free radical iron injury and this circumstance is somewhat similar to intracranial bleeding [23].

We confirmed that women underwent decompressive craniectomy less often than men even though decompressive craniectomy was associated with increased survival in both sexes in the multivariable regression analysis. Our group formerly described increasing rates for this procedure during the period 2003–2012 in our country [24]. Most research work in this regard comes from uncontrolled studies, which show modest beneficial effects after craniectomy and evacuation of a hematoma [25]. Its apparent association with a better outcome may be confounded by the indication of the procedure in patients with better functional condition or health status before the stroke, or rather the subjective perception on behalf of the treating clinicians of a better prognosis after the surgery. A randomized controlled trial to determine the actual usefulness of decompressive craniectomy in the management of HS is currently ongoing [26].

Obesity was associated with a lower IHM both among women and men. The design of our study does not allow us to know the answer to what has been called the obesity paradox in stroke [27]. Regrettably, many of the studies that deal with this concept have an observational study design, show inaccuracies in body weight measurement and are susceptible to selection and survival biases too [28].

We agree with many other observational and clinical studies finding increased IHM after HS among those taking OACs and antiplatelet agents prior to hospitalization besides the influence of sex [29,30,31,32,33,34].

Inohara et al. analyzed data of 141,311 patients with HS and reported that after adjustment for confounders, prior use of warfarin (OR 1.62; 97.5% CI 1.53–1.71) and prior use of non–vitamin K antagonist oral anticoagulants (OR, 1.21; 97.5% CI 1.11–1.32) were associated with increased IHM when compared with those not taking OACs. These associations remained significant when patients using any antiplatelet agents were excluded [29].

Data of 13,291 patients included in the Swedish Stroke Register between 2012 and 2016 showed that OACs (hazard ratio 1.93; 95% CI, 1.57–2.38) and antiplatelet agents (hazard ratio 1.32; 95% CI, 1.13–1.54) were associated with higher risk of mortality in the first 24 h [30].

A meta-analysis including results of 25 cohort studies concluded that IHM in patients with preceding use of antiplatelet agent was 27% higher than among those not consuming these drugs (OR 1.27, 95% CI 1.10–1.47) [31].

The larger baseline hematoma and the increased risk of hematoma expansion are the reasons for the negative effect of OACs and antiplatelet agents on severity and mortality after HS [29,30,31,32,33,34]. Unfortunately, in the SNHDD, information regarding the initial hematoma volume, degree of hematoma expansion and type, duration or number of OACs and antiplatelet agents used is not available [11,24].

In our multivariable regression analysis, women were more likely than men to die in the hospital when admitted for HS. Again, differences in the design of the studies can offer variable results [35]. If it is true that women have a poorer short-term prognosis after a HS and the differences cannot be explained by a distinct medical management, biology could eventually provide the answer. Inflammation and cell death might occur earlier, as seen in animal models of intracranial hemorrhages induced by bacterial collagenases [36]. The mechanical injury of the brain tissue and its direct consequence, brain edema, the potential reparative and anti-inflammatory roles of microglia and macrophages in the later phase of the intracranial hemorrhage to resorb the bleeding and resolve the edema, or the release of thrombin, plasmin and reactive oxygen species that cause brain cell damage following intracranial hemorrhage, might differ between sexes [37,38,39].

In addition to those mentioned before, several limitations must be considered. First, we lack data on baseline stroke severity, which is an important predictor of outcome and, therefore, an important residual confounder variable [24,40,41,42,43]. Second, a selection bias is possible because HS patients having more rapidly fatal and less severe symptoms and those with a worse access to medical care may be underrepresented [40]. Third, in the SNHDD, information on laboratory results is not collected, so the effect of hematological or biochemical disorders cannot be evaluated [11]. Fourth, even if PSM has surely helped to attenuate differences in baseline clinical variables, complete elimination of residual confounding is difficult to achieve in observational studies [40,41,42,43]. Fifth, as we used anonymized data, a patient hospitalized two or more times with HS would be considered as different individuals [11]. Sixth, we excluded from the study population patients with a code for history of previous stroke. Therefore, this condition was not analyzed as a factor associated with IHM. Seventh, the quality of coding may be affected by poor charting by physicians, lack of time to “code everything”, lack of perceived importance by health coders or when codes that lead to greater reimbursement of funds to hospitals are favored over the principal reason for which a patient is hospitalized [24,40,41,43]. Eighth, mortality once patients are discharged from the hospital is not available [11]. Ninth, stratifying patients according to the intracerebral hemorrhage (ICH) score as well as the Hunt–Hess grade for subarachnoid hemorrhage (SAH) would have improved the results of our investigation [44,45]. Unfortunately, the SNHDD does not include data on the ICH volume or the Glasgow Coma Scale coma severity score necessary to calculate the ICH score. Furthermore, detailed symptomatology, such as severity of headache or neck stiffness, among others, that are used to estimate the Hunt–Hess grade for SAH are also lacking in the SNHDD.

However, the strengths of administrative databases such as the SNHDD, include that (1) they reflect real-world practice at a national level; (2) they are large, so infrequent diseases or risk factors associated with stroke can also be evaluated; (3) they are a cost-effective alternative for monitoring stroke incidence and burden; (4) the validity of ICD codes to investigate HS in administrative databases has been demonstrated in Spain and other countries suggesting that when these data are applied to an appropriate question with validated case definitions, high-quality and reliable conclusions can be inferred [40,41,42,43,46,47,48,49,50]. In a representative sample of 30 Spanish hospitals, when compared with medical records, the ICD-9 codes for HS as a primary diagnosis registered in the SNHDD were correct in 43 of 49 cases reviewed (87.75%) [50].

In our opinion, the strengths of this study and its uniqueness clearly outweigh its limitations. Furthermore, the SNHDD, like hospital discharge administrative databases from other countries, has already been used to assess trends, demographic and clinical characteristics and outcomes of hospitalizations with stroke [24,51,52,53,54,55,56,57].

In any case, our study is the starting point for future investigations that should confirm or discard our conclusions using larger and more precise clinical data.

## 5. Conclusions

In conclusion, women were more likely than men to die in the hospital when admitted for HS even accounting for confounding factors through a matched-pair analysis. Although biological differences are a possibility, especially after menopause, the scientific community should be concerned about plausible disparities in the clinical management based on patients’ sex, such as a lesser use of potentially lifesaving procedures.

## Figures and Tables

**Table 1:** Incidence of hospitalizations for hemorrhagic stroke in Spain from 2016 to 2018 according to sex and age groups.

	Women	Men	
**Age Groups**	***n* (Inc/10^5^ Women)**	***n* (Inc/10^5^ Men)**	***p*** **-Value**
18–54 years	3787 (10.8)	5134 (14.5)	<0.001
55–69 years	4948 (38.9)	8256 (68.8)	<0.001
70–84 years	10,540 (123.7)	13,469 (201.8)	<0.001
≥85 years	6084 (213.0)	5009 (346.3)	<0.001
All age groups	25,359 (43.0)	31,868 (57.3)	<0.001

Inc/10^5^: Incidence per 100,000 inhabitants. The *p*-values are for comparisons between men and women.

**Table 2:** Type of hemorrhagic stroke, age, Charlson comorbidity index, cardiovascular risk factors and alcohol abuse before and after propensity score matching (PSM) for men and women hospitalized for hemorrhagic stroke in Spain from 2016 to 2018.

Variables	Before PSM			After PSM		
	Women	Men	*p*-Value	Women	Men	*p*-Value
Nontraumatic subarachnoid hemorrhage, *n* (%)	6382 (25.2)	4642 (14.6)	<0.001	6382 (25.2)	4422 (17.44)	<0.001
Nontraumatic intracerebral hemorrhage, *n* (%)	13,730 (54.1)	17,759 (55.7)	<0.001	13,730 (54.1)	14,326 (56.5)	<0.001
Other and unspecified nontraumatic intracranial hemorrhage, *n*(%)	5247 (20.7)	9467 (29.7)	<0.001	5247 (20.7)	6611 (26.1)	<0.001
Age, mean (SD)	72.74 (15.1)	70.20 (14.4)	<0.001	72.74 (15.1)	71.50 (13.8)	<0.001
18–54 years, *n* (%)	3787 (14.9)	5134 (16.1)	<0.001	3787 (14.9)	3523 (13.9)	<0.001
55–69 years, *n* (%)	4948 (19.5)	8256 (25.9)	<0.001	4948 (19.5)	6291 (24.8)	<0.001
70–84 years, *n* (%)	10,540 (41.6)	13,469 (42.3)	0.091	10,540 (41.6)	11,063 (43.6)	<0.001
≥85 years, *n* (%)	6084 (24.0)	5009 (15.7)	<0.001	6084 (24.0)	4482 (17.7)	<0.001
CCI, mean (SD)	0.67 (0.7)	0.85 (0.76)	<0.001	0.67 (0.7)	0.63 (0.6)	<0.001
CCI = 0, *n* (%)	13,316 (52.5)	14,202 (44.6)	<0.001	13,316 (52.5)	13,449 (53.0)	0.237
CCI = 1, *n* (%)	8224 (32.4)	10,856 (34.1)	<0.001	8224 (32.4)	8600 (33.9)	<0.001
CCI > 1, *n* (%)	3819 (15.1)	6810 (21.4)	<0.001	3819 (15.1)	3310 (13.1)	<0.001
Obesity, *n* (%)	1377 (5.4)	1631 (5.1)	0.097	1377 (5.4)	1159 (4.6)	<0.001
Hypertension, *n* (%)	13,134 (51.8)	17,045 (53.5)	<0.001	13,134 (51.8)	13,548 (53.4)	<0.001
Lipid metabolism disorders, *n* (%)	7374 (29.1)	9453 (29.7)	0.127	7374 (29.1)	7092 (28.0)	0.006
Alcohol abuse, *n* (%)	411 (1.6)	2971 (9.3)	<0.001	411 (1.6)	1982 (7.8)	<0.001
Use of oral anticoagulants, *n* (%)	3478 (13.71)	4404 (13.82)	0.718	3478 (13.71)	3467 (13.67)	0.887
Use of antiplatelet agents, *n* (%)	1983 (7.82)	3211 (10.08)	<0.001	1983 (7.82)	2002 (7.89)	0.754

PSM: propensity score matching. SD: standard deviation. CCI: Charlson comorbidity index. The *p*-values are for comparisons between men and women.

**Table 3:** Comorbidities, use of therapeutic procedures and hospital outcomes before and after propensity score matching (PSM) for men and women hospitalized for hemorrhagic stroke in Spain from 2016 to 2018.

Variables	Before PSM			After PSM		
	Women	Men	*p*-Value	Women	Men	*p*-Value
Acute myocardial infarction, *n* (%)	320 (1.3)	1085 (3.4)	<0.001	320 (1.3)	144 (0.6)	<0.001
Congestive heart failure, *n* (%)	1020 (4.0)	1237 (3.9)	0.391	1020 (4.0)	922 (3.6)	0.023
Peripheral vascular disease, *n* (%)	399 (1.6)	1230 (3.9)	<0.001	399 (1.6)	225 (0.9)	<0.001
Dementia, *n* (%)	1916 (7.6)	1390 (4.4)	<0.001	1916 (7.6)	1314 (5.2)	<0.001
COPD, *n* (%)	1340 (5.3)	3016 (9.5)	<0.001	1340 (5.3)	1198 (4.7)	0.004
Rheumatoid disease, *n* (%)	408 (1.6)	232 (0.7)	<0.001	408 (1.6)	229 (0.9)	<0.001
Peptic ulcer, *n* (%)	55 (0.2)	138 (0.4)	<0.001	55 (0.2)	49 (0.2)	0.556
Diabetes, *n* (%)	4475 (17.7)	7449 (23.4)	<0.001	4475 (17.7)	4884 (19.3)	<0.001
Hemiplegia/Paraplegia, *n* (%)	4009 (15.8)	5474 (17.2)	<0.001	4009 (15.8)	4097 (16.2)	0.286
Chronic renal disease, *n* (%)	1615 (6.4)	2619 (8.2)	<0.001	1615 (6.4)	1612 (6.4)	0.956
Chronic liver disease, *n* (%)	777 (3.1)	1670 (5.2)	<0.001	777 (3.1)	778 (3.1)	0.979
Cancer, *n* (%)	440 (1.7)	1113 (3.5)	<0.001	440 (1.7)	344 (1.4)	0.001
Metastatic cancer, *n* (%)	251 (1.0)	448 (1.4)	<0.001	251 (1.0)	269 (1.1)	0.428
AIDS, *n* (%)	21 (0.9)	94 (0.3)	<0.001	21 (0.9)	5 (0.0)	0.002
Atrial fibrillation, *n* (%)	4644 (18.3)	5715 (17.9)	0.241	4644 (18.3)	4533 (17.9)	0.200
Anemia, *n* (%)	726 (2.9)	626 (2.0)	<0.001	726 (2.9)	563 (2.2)	<0.001
Depression, *n* (%)	1928 (7.6)	960 (3.0)	<0.001	1928 (7.6)	938 (3.7)	<0.001
Sepsis, *n* (%)	254 (1.0)	462 (1.5)	<0.001	254 (1)	355 (1.4)	<0.001
Nosocomial pneumonia, *n* (%)	421 (1.7)	684 (2.2)	<0.001	421 (1.7)	511 (2.0)	0.003
Mechanical ventilation, *n* (%)	3227 (12.7)	4034 (12.7)	0.811	3227 (12.7)	3044 (12)	0.014
Decompressive craniectomy, *n* (%)	1262 (5.0)	2052 (6.4)	<0.001	1262 (5.0)	1578 (6.2)	<0.001
LOHS, median (IQR)	8 (13)	7 (12)	<0.001	8 (13)	7 (12)	<0.001
In-hospital mortality, *n* (%)	7344 (29.0)	7719 (24.2)	<0.001	7344 (29.0)	6019 (23.7)	<0.001

PSM: propensity score matching. COPD: chronic obstructive pulmonary disease. AIDS acquired immunodeficiency syndrome. LOHS: length of hospital stay. IQR: interquartile range. The *p*-values are for comparisons between men and women.

**Table 4:** In-hospital mortality according to type of hemorrhagic stroke, age, Charlson comorbidity index and lifestyle variables before and after propensity score matching (PSM) for men and women hospitalized for hemorrhagic stroke in Spain from 2016 to 2018.

Variables	IHM Before PSM			IHM After PSM		
	Women	Men	*p*-Value	Women	Men	*p*-Value
Nontraumatic subarachnoid hemorrhage, *n* (%)	1478 (23.2)	986 (21.2)	0.017	1478 (23.2)	912 (20.6)	0.002
Nontraumatic intracerebral hemorrhage, *n* (%)	4598 (33.5)	5180 (29.2)	<0.001	4598 (33.5)	4137 (28.9)	<0.001
Other and unspecified nontraumatic intracranial hemorrhage, *n* (%)	1268 (24.2)	1553 (16.4)	<0.001	1268 (24.2)	970 (14.7)	<0.001
Age, mean (SD)	77.55 (12.9)	74.00 (13.3)	<0.001	77.55 (12.9)	75.48 (12.6)	<0.001
18–54 years, *n* (%)	544 (14.4)	799 (15.6)	0.118	544 (14.4)	490 (13.9)	0.576
55–69 years, *n* (%)	1029 (20.8)	1591 (19.3)	0.033	1029 (20.8)	1147 (18.2)	0.001
70–84 years, *n* (%)	3280 (31.1)	3548 (26.3)	<0.001	3280 (31.1)	2802 (25.3)	<0.001
≥85 years, *n* (%)	2491 (40.9)	1781 (35.6)	<0.001	2491 (40.9)	1580 (35.3)	<0.001
CCI, mean (SD)	0.76 (0.7)	1.03 (0.9)	<0.001	0.76 (0.7)	0.77 (0.7)	0.665
CCI = 0, *n* (%)	3484 (26.2)	2856 (20.1)	<0.001	3484 (26.2)	2747 (20.4)	<0.001
CCI = 1, *n* (%)	2539 (30.9)	2716 (25.0)	<0.001	2539 (30.9)	2204 (25.6)	<0.001
CCI > 1, *n* (%)	1321 (34.6)	2147 (31.5)	0.001	1321 (34.6)	1068 (32.3)	0.038
Obesity, *n* (%)	404 (29.3)	367 (22.5)	<0.001	404 (29.3)	267 (23.0)	<0.001
Hypertension, *n* (%)	3885 (29.6)	3976 (23.3)	<0.001	3885 (29.6)	3098 (22.9)	<0.001
Lipid metabolism disorders, *n* (%)	2123 (28.8)	2223 (23.5)	<0.001	2123 (28.8)	1625 (22.9)	<0.001
Alcohol abuse, *n* (%)	111 (27.0)	703 (23.7)	0.137	111 (27.0)	443 (22.4)	0.042
Use of oral anticoagulants, *n* (%)	1131 (38.27)	1390 (31.56)	<0.001	1131 (38.27)	1267 (36.42)	0.111
Use of antiplatelet agents, *n* (%)	692 (34.89)	965 (30.10)	0.001	692 (34.89)	1065 (33.17)	0201

IHM: in-hospital mortality. PSM: propensity score matching. SD: standard deviation. CCI: Charlson comorbidity index. The *p*-values are for comparisons between men and women.

**Table 5:** In-hospital mortality (IHM) according to comorbidities, use of therapeutic procedures and length of hospital stay before and after propensity score matching (PSM) for men and women hospitalized for hemorrhagic stroke in Spain from 2016 to 2018.

Variables	IHM Before PSM			IHM After PSM		
	Women	Men	*p*-Value	Women	Men	*p*-Value
Acute myocardial infarction, *n* (%)	122 (38.1)	339 (31.2)	0.021	122 (38.1)	51 (35.4)	0.577
Congestive heart failure, *n* (%)	422 (41.4)	485 (39.2)	0.297	422 (41.4)	354 (38.4)	0.181
Peripheral vascular disease, *n* (%)	113 (28.3)	381 (31.0)	0.316	113 (28.3)	80 (35.6)	0.061
Dementia, *n* (%)	748 (39.0)	497 (35.8)	0.054	748 (39.0)	471 (35.8)	0.066
COPD, *n* (%)	406 (30.3)	900 (29.8)	0.761	406 (30.3)	387 (32.3)	0.277
Rheumatoid disease, *n* (%)	113 (27.7)	57 (24.6)	0.389	113 (27.7)	56 (24.5)	0.374
Peptic ulcer, *n* (%)	12 (21.8)	34 (24.6)	0.678	12 (21.8)	15 (30.6)	0.309
Diabetes, *n* (%)	1471 (32.9)	1970 (26.5)	<0.001	1471 (32.9)	1251 (25.6)	<0.001
Hemiplegia/Paraplegia, *n* (%)	1032 (25.7)	1255 (22.9)	0.002	1032 (25.7)	930 (22.7)	0.001
Chronic renal disease, *n* (%)	599 (37.1)	901 (34.4)	0.076	599 (37.1)	537 (33.3)	0.025
Chronic liver disease, *n* (%)	273 (35.1)	543 (32.5)	0.201	273 (35.1)	257 (33.0)	0.382
Cancer, *n* (%)	154 (35.0)	399 (35.9)	0.753	154 (35.0)	137 (39.8)	0.165
Metastatic cancer, *n* (%)	123 (49.0)	193 (43.1)	0.131	123 (49.0)	122 (45.4)	0.405
AIDS, *n* (%)	9 (42.9)	30 (31.9)	0.341	9 (42.9)	2 (40.0)	0.908
Atrial fibrillation, *n* (%)	1821 (39.2)	1991 (34.8)	<0.001	1821 (39.2)	1575 (34.8)	<0.001
Anemia, *n* (%)	204 (28.1)	151 (24.1)	0.098	204 (28.1)	129 (22.9)	0.035
Depression, *n* (%)	493 (25.6)	228 (23.8)	0.287	493 (25.6)	220 (23.5)	0.219
Sepsis, *n* (%)	135 (53.2)	244 (52.8)	0.931	135 (53.2)	180 (50.7)	0.552
Nosocomial pneumonia, *n* (%)	134 (31.8)	223 (32.6)	0.790	134 (31.8)	159 (31.1)	0.815
Mechanical ventilation, *n* (%)	1896 (58.8)	2397 (59.4)	0.566	1896 (58.8)	1768 (58.1)	0.589
Decompressive craniectomy, *n* (%)	262 (20.8)	321 (15.6)	<0.001	262 (20.8)	225 (14.3)	<0.001
LOHS, median (IQR)	3 (7)	3 (7)	0.897	3 (7)	3 (7)	0.766

IHM: in-hospital mortality. PSM: propensity score matching. COPD: chronic obstructive pulmonary disease. AIDS acquired immunodeficiency syndrome. LOHS: length of hospital stay. IQR: interquartile rate. The *p*-values are for comparisons between men and women.

**Table 6:** Multivariable logistic regression analysis of factors associated with in-hospital mortality for both women and men hospitalized for hemorrhagic stroke in Spain from 2016 to 2018 and sensitivity analysis combining data from women and men.

	Women	Men	Both
**Variables**	**OR (95% CI)**	**OR (95% CI)**	**OR (95% CI)**
18–54 years	1	1	1
55–69 years	1.67 (1.48–1.90)	1.38 (1.24–1.53)	1.57 (1.43–1.72)
70–84 years	4.02 (3.58–4.53)	2.80 (2.53–3.10)	3.45 (3.17–3.76)
≥85 years	7.73 (6.82–8.76)	5.38 (4.80–6.04)	6.72 (6.13–7.36)
Obesity	0.87 (0.82–0.93)	0.88 (0.83–0.93)	0.88 (0.84–0.92)
Congestive heart failure	1.26 (1.10–1.45)	1.45 (1.27–1.65)	1.33 (1.20–1.47)
Peripheral vascular disease	NS	1.20 (1.05–1.38)	NS
Dementia	1.41 (1.27–1.56)	1.62 (1.44–1.83)	1.51 (1.40–1.64)
COPD	NS	1.17 (1.04–1.41)	NS
Diabetes	1.10 (1.02–1.19)	1.09 (1.02–1.16)	1.06 (1.01–1.13)
Chronic renal disease	NS	1.34 (1.21–1.48)	1.18 (1.08–1.29)
Sepsis	2.50 (1.88–3.32)	2.83 (2.28–3.51)	2.58 (2.15–3.11)
Use of oral anticoagulants	1.64(1.41–186)	1.59(1.30–1.70)	1.62(1.35–1.80)
Use of antiplatelet agents	1.36 (1.18–1.60)	1.31(1.11–1.52)	1.33 (1.15–1.51)
Mechanical ventilation	9.63 (8.77–10.59)	11.61 (10.68-12.63)	10.51 (9.82–11.24)
Decompressive craniectomy	0.49 (0.42–0.58)	0.38 (0.33–0.43)	0.41 (0.36–0.46)
Women	NA	NA	1.23 (1.18–1.28)

OR (95% CI): odds ratio (95% confidence interval). COPD: chronic obstructive pulmonary disease. NS: non-significant (only variables with significant results in the multivariable regression analysis are shown in the table). NA: not applicable.

## Data Availability

According to the contract signed with the Spanish Ministry of Health and Social Services, which provided access to the databases from the Spanish National Hospital Database (Conjunto Mínimo Basico de Datos; CMBD), we cannot share the databases with any other investigator, and we have to destroy the databases once the investigation has concluded. Consequently, we cannot upload the databases to any public repository. However, any investigator can apply for access to the databases by filling out the questionnaire available at http://www.msssi.gob.es/estadEstudios/estadisticas/estadisticas/estMinisterio/SolicitudCMBDdocs/Formulario_Peticion_Datos_CMBD.pdf. All other relevant data are included in the paper.

## Supplementary Materials

The following are available online at https://www.mdpi.com/article/10.3390/jcm10163753/s1, Table S1: International Classification of Disease, 10th edition, (ICD-10) codes for the clinical diagnoses and procedures used in this investigation.
