# Supplementary material for: Incidence and Outcomes of Hemorrhagic Stroke among Adults in Spain (2016–2018) According to Sex: A Retrospective, Cohort, Observational, Propensity Score Matched Study
Source: J Clin Med. 2021 Aug 23;10(16):3753. doi: 10.3390/jcm10163753 (PMC8397207; doi:10.3390/jcm10163753)
Supplement: Supplementary file 1 [file jcm-10-03753-s001.zip › jcm-1313995-supplementary.pdf]

Table S1. International Classification of Disease, 10th edition, (ICD-10) codes for the clinical diagnoses and procedures used in this investigation.

Clinical diagnoses and procedures	ICD-10 codes
Nontraumatic subarachnoid hemorrhage	I60.0, I60.00; I60.01; I60.02; I60.1; I60.10, I60.11, I60.12, I60.2, I60.3; I60.30, I60.31, I60.32, I60.4, I60.5, I60.50, I60.51, I60.52, I60.6, I60.7, I60.8, I60.9
Nontraumatic intracerebral hemorrhage	I61.0, I61.2, I61.3, I61.4, I61.5, I61.6, I61.8, I61.9
Other and unspecified nontraumatic intracranial hemorrhage	I62.0, I62.00, I62.01, I62.02, I62.03, I62.1, I62.9
Obesity	E66.X
Hypertension	I10, I16.6
Lipid metabolism disorders	E78.0X-E78.5
Alcohol abuse	F10, E52, G62.1, I42.6, K29.2, K70.0, K70.3, K70.9, T51.x, Z50.2, Z71.4, Z72.1
Atrial fibrillation	I48.0, I48.1, I48.2, I48.91
Anemia	D50.0, D50.8, D50.9, D51.x-D53.x
Depression	F20.4, F31.3-F31.5, F32.x, F33.x, F34.1, F41.2, F43.2
Sepsis	R65.20, R65.21, A40.X, A41.XX
Nosocomial pneumonia	J12-J18, J95.851
Use of oral anticoagulants	Z79.01
Use of antiplatelet agents	Z79.02, Z79.82
Mechanical ventilation	5A1945Z, 5A1955Z, 5A1935Z,5A09357, 5A09457, 5A09557
Decompressive craniectomy	00J00ZZ, 00W00JZ, 00W00KZ, 0N800ZZ, 0N803ZZ, 0N804ZZ, 0NC10ZZ, 0NC13ZZ, 0NC14ZZ, 0NC30ZZ, 0NC33ZZ, 0NC34ZZ, 0NC40ZZ, 0NC43ZZ, 0NC44ZZ, 0NC50ZZ, 0NC53ZZ, 0NC54ZZ, 0NC60ZZ, 0NC63ZZ, 0NC64ZZ, 0NC70ZZ, 0NC73ZZ, 0NC74ZZ, 0NH00MZ, 0NH03MZ, 0NH04MZ, 0NP000Z, 0NP004Z, 0NP005Z, 0NP007Z, 0NP007Z, 0NP00KZ, 0NP00SZ, 0NP030Z, 0NP034Z, 0NP037Z, 0NP03KZ, 0NP03SZ, 0NP040Z, 0NP044Z, 0NP047Z, 0NP04KZ, 0NP04SZ, 0NP0X4Z, 0NP0XSZ, 0NW000Z, 0NW004Z, 0NW005Z, 0NW007Z, 0NW00JZ, 0NW00KZ, 0NW00MZ, 0NW00SZ, 0NW030Z, 0NW034Z, 0NW035Z, 0NW037Z, 0NW03JZ, 0NW03KZ, 0NW03MZ, 0NW03SZ, 0NW040Z, 0NW044Z, 0NW045Z, 0NW047Z, 0NW04JZ, 0NW04KZ, 0NW04MZ, 0NW04SZ, 0W9100Z, 0W010ZZ, 0W9130Z, 0W913ZZ, 0W9140Z, 0W914ZZ, 0WC10ZZ, 0WC13ZZ, 0WC14ZZ, 0WH10YZ, 0WH13YZ, 0WH14YZ, 0WJ10ZZ, 0WP100Z, 0WP101Z, 0WP10JZ, 0WP10YZ, 0WP130Z, 0WP131Z, 0WP13JZ, 0WP13YZ, 0WP140Z,

Clinical diagnoses and procedures	ICD-10 codes
	0WP141Z, 0WP14JZ, 0WP14HZ, 0WW00Z, 0WW101Z, 0WW103Z, 0WW10JZ, 0WW10YZ, 0WW130Z, 0WW131Z, 0WW133Z, 0WW13JZ, 0WW13YZ, 0WW140Z, 0WW141Z, 0WW143Z, 0WW14JZ, 0WW14YZ, 0N500ZZ, 0N503ZZ, 0N504ZZ, 0NB00ZZ, 0NB03ZZ, 0NB04ZZ, 0NT10ZZ, 0NT30ZZ, 0NT40ZZ, 0NT50ZZ, 0NT60ZZ, 0NT70ZZ, 009100Z, 00910ZZ, 00C10ZZ, 00C13ZZ, 00C14ZZ, 009000Z, 00900ZZ, 009030Z, 00903ZZ, 009040Z, 00904ZZ, 00C00ZZ, 00C03ZZ, 00C04ZZ, 00H003Z, 00H003Z, 00H00YZ, 00H032Z, 00H033Z, 00H03YZ, 00H042Z, 00H043Z, 00H04YZ, 00H602Z, 00H603Z, 00H60YZ, 00H632Z, 00H633Z, 00H63YZ, 00H642Z, 00H643Z, 00H64YZ, 00P000Z, 00P002Z, 00P003Z, 00P007Z, 00P00JZ, 00P00KZ, 00P00YZ, 00P030Z, 00P032Z, 00P033Z, 00P037Z, 00P03JZ, 00P03KZ, 00P03YZ, 00P040Z, 00P042Z, 00P043Z, 00P047Z, 00P04JZ, 00P04KZ, 00P04YZ, 00P600Z, 00P602Z, 00P603Z, 00P60YZ, 00P630Z, 00P632Z, 00P633Z, 00P63YZ, 00P640Z, 00P642Z, 00P643Z, 00P64YZ, 00P6X2Z, 00W000Z, 00W002Z, 00W003Z, 00W007Z, 00W00MZ, 00W00YZ, 00W030Z, 00W032Z, 00W033Z, 00W037Z, 00W03JZ, 00W03KZ, 00W03MZ, 00W03YZ, 00W040Z, 00W042Z, 00W043Z, 00W047Z, 00W04JZ, 00W04KZ, 00W04MZ, 00W04YZ, 00W600Z, 00W602Z, 00W603Z, 00W60MZ, 00W60YZ, 00W630Z, 00W632Z, 00W633Z, 00W63MZ, 00W63YZ, 00W640Z, 00W642Z, 00W643Z, 00W64MZ, 00W64YZ, 00B70ZZ, 00B73ZZ, 00B74ZZ, 0500ZZ, 00503ZZ, 00504ZZ, 00B00ZZ, 00B03ZZ, 00B04ZZ.
